# News and Coming Events

**Published:** 1909-06-05

**Authors:** 


### News and Coming Events.

Dr. Georges Dreyer, M.A.Oxon, M.D. Copenhagen, Professor of Pathology in the University of Oxford, has been elected a member of the Danish Royal Academy of Letters and Science.

Dr. Edward Liveing, M.D.Cantab., F.R.C.P., has resigned the registrarship of the Royal College of Physicians of London, which he has held for the last twenty years. The Council has placed on record its appreciation of the devotion and energy with which Dr. Liveing discharged the duties of his office during so many years.

The Annual Welsh Medical Dinner will be held at the Criterion Restaurant, London, on Tuesday, June 15, when the chair will be taken by Dr. D. C. Lloyd Owen, of Birmingham, at 7.30 p.m. The date has been selected for the convenience of those who will be visiting London for the National Eisteddfod.

Mr. Grafton Elliot Smith, Professor of Anatomy in the Government School of Medicine, Cairo, since 1900, has been appointed by Manchester University to the Chair of Anatomy, which has been held for the last 24 years by Professor Young, lately resigned. Mr. Smith was educated at the University of Sydney, New South Wales, and qualified in 1893. He was appointed successively Demonstrator in Anatomy and King Travelling Fellowship in the University of Sydney. Proceeding to England, he continued his anatomical research work at Cambridge, and obtained a Fellowship of St. John's College, and a University Demonstratorship of Anatomy.

The fourteenth annual meeting of the Guy's Hospital Ladies' Association was held last week under the presidency of Princess Christian of Schleswig-Holstein. Steady progress and an improved financial position were reported, and the zeal of the twenty-two branches in sending several hundreds of garments was commended. The Victoria Ward of eight beds, maintained by the Association, has just been completed and is now ready for occupation, and the junior branch is being reorganised with a view to the maintenance of a cot by juvenile supporters. Miss Swift, the matron, gratefully acknowledged the help rendered by the Association, and Mr. Cosmo Bonsor, the treasurer, dealt with the training of nurses at Guy's Hospital.

A dispute, with reference to "Ancient Lights," which has arisen between the Governors of Westminster Hospital and the trustees of the Wesleyan Twentieth Century Fund, who are erecting a memorial hall on the site of the old Aquarium, recently came before Mr. Justice Joyce in the Chancery Division. Mr. Slater, the architect appointed by the judge, reported that if the proposed plans for the hall are carried out the access of light to a number of wards in the hospital will be considerably interfered with, and the recovery of patients would thereby be prejudiced and retarded. The Hospital Governors, through Mr. Hughes, K.C., asked for a speedy trial of the action, and in the meanwhile for an injunction restraining the defendants from building to a greater height than that of the demolished Aquarium. The defendants challenged Mr. Slater's report, claiming the right, enjoyed by the last owners, to build to a height of 52 feet, and they asked that the case should stand over until the Michaelmas Sittings. His Lordship granted the injunction, adding that an early trial was clearly desirable.

A Meeting of the Socialist Medical League was held under the chairmanship of Dr. Salter, L.C.C., at the Holborn Restaurant, London, on June 3, when Dr. J. J. Robb opened a discussion on The Nationalisation of the Medical Profession.

The members of the Council of the Royal College of Surgeons of England who retire this year by rotation are Mr. A. W. Mayo Robson, Sir W. Watson Cheyne, and Mr. R. Clement Lucas. It is understood that Mr. Harrison Cripps and Mr. W. W. H. Jessop, senior surgeon and senior ophthalmic surgeon respectively at St. Bartholomew's Hospital, intend to offer themselves as candidates at the forthcoming annual election. The list of nominations for election to the Council closed on Friday of this week.

The British League against Tuberculosis has arisen out of the recent National Tuberculosis Conference at Caxton Hall. A large number of influential names are published in support of the League, and many medical men are upon the Advisory and Executive Committees, while Sir Thomas Oliver, M.D., is a Vice-President. The main objects of the League, which will shortly inaugurate branches in the principal industrial and rural districts of the United Kingdom, are, briefly, to instruct the public generally in the war against tuberculosis, on the main lines of scientific prevention and treatment, by means of lectures and demonstrations, and to arouse public opinion on the dangers of the communicability of tuberculosis from animals to man through diseased meat and milk. Special efforts will be devoted to active propaganda through educational and local governing bodies.

### THE TUBERCULOSIS EXHIBITION AND CONGRESS.

The National Association for the Prevention of Consumption and other Forms of Tuberculosis is now holding a free Tuberculosis Exhibition for the education of the public, and more especially its poorer sections, at the Art Gallery, Whitechapel, and it will remain open until June 19. On June 2 the opening address was delivered by the President of the Local Government Board, Right Hon. John Burns, M.P., and on June 8, 9, and 10 a Conference will be held, beginning at 11 a.m. each day. June 8 will be devoted to "The Detection of Consumption," and papers will be read by Dr. Scurfield, Medical Officer of Health, Sheffield; Dr. Philip, Physician to the Royal Victoria Hospital for Consumption, Edinburgh; and Miss Cummins, Lady Almoner, St. Thomas's Hospital. "The Treatment of Consumption" will occupy June 9, and Dr. Arthur Latham, Physician to St. George's Hospital; Dr. Paterson, Superintendent, Frimley Sanatorium; Dr. Walker, Medical Superintendent, East Anglian Sanatorium, Nayland; and Dr. H. W. McConnel will read papers. June 10 will be devoted to "The Prevention of Consumption," and the papers will be read by Dr. Duncan Forbes, Medical Officer of Health, Brighton; Dr. E. W. Hope, Medical Officer of Health, Liverpool; and Dr. J. Edwin Squire, C.B., Senior Physician to Mount Vernon Hospital for Consumption; while at 3 p.m. a demonstration of meat inspection will be given by Mr. J. King, M.R.C.V.S., Chief Veterinary Inspector, Metropolitan Cattle Market. Evening lectures, at 8 p.m., form a feature of the exhibition, and the lecturers include: Dr. C. Theodore Williams, Dr. Frederick Rose, Dr. Newsholme, Medical Officer to the Local Government Board, Professor G. Sims Woodhead, Dr. Hector Mackenzie, and Dr. Nathan Raw.

WITH a view to raising part of the sum of £95,000 required to complete the removal fund of King's College Hospital, a carnival is to be held at the Crystal Palace on July 1. Mr. George Heyer, the Appeal Secretary, has ordered 300,000 tickets, and he now hopes to order 1,000,000, since the South-Eastern and Chatham and the Brighton offer return tickets to London on July 1 at one fare and a quarter to holders of shilling carnival tickets.

THE annual meeting of the Birmingham Medical Benevolent Society was held at the Midland Hotel on May 25. Dr. Malet, the retiring president, occupied the chair, and was supported, among others, by Sir James Sawyer and Sir Thomas Chavasse. The eighty-seventh annual report states that the invested funds of the society amount to £15,940, with a balance of £120 9s. 7d. at the bank. The annual value of the grants ranges from £18 to £36, and the sum expended in this way during 1908 was £757. The total number of benefit members of the society at the end of the year was 390. The chairman moved the adoption of the report, and Sir James Sawyer, in seconding the resolution, complained of the apathy of members of the profession towards the society. Notwithstanding that there are about 4,000 medical men in the area covered by the society only 400 are members of the organisation. Sir Thomas Chavasse was elected president and Dr. Simon president-elect. A motion by Dr. Thomas Wilson to amend the rules of the society so as to admit as members lady practitioners was carried.

IN connection with the annual grant voted by Parliament in aid of scientific investigations concerning the causes and processes of disease, the President of the Local Government Board has authorised the following special researches: (1) A continuation of the investigation into protracted and recurrent infection in enteric fever, by Dr. Theodore Thomson, Medical Inspector of the Board, in conjunction with Dr. Hedingham, of the Lister Institute. (2) A continuation of the investigation into protracted and recurrent infection in diphtheria, by Dr. Theodore Thomson and Dr. C. J. Thomas. (3) A continuation of the investigation into flies as carriers of infection by Dr. Monckton Copeman, Medical Inspector of the Board, and by Professor Nuttall, of Cambridge. (4) A continuation of Dr. Andrewes's investigation on the presence of sewage bacteria in sewer air, with a view to ascertaining their number and the distance they can be carried by air currents. Also a continuation of Dr. Andrewes's investigation into the part played by changes in bone marrow in the defensive mechanism of the body against infection. (5) A continuation of Dr. Savage's investigations on the bacterial measurement of milk pollution, and on the presence of the Gaertner group of bacilli in prepared meats and allied foods. (6) An investigation into the chemical and physical changes undergone by milk as the result of infection by bacteria, and into the relation of the pancreas to epidemic diarrhœa, by Dr. Schölberg and Mr. Wallis, of University College, Cardiff. (7) An investigation of the records of charitable lying-in hospitals as to the nutrition of the mother and other factors influencing the vitality of infants and their progress in the first 14 days of life, by Dr. Darwall Smith, physician to the British Lying-in Hospital. (8) An investigation into the occurrence and importance, in relation to treatment, of mixed infections in pulmonary tuberculosis, by Dr. Inman, pathologist to the Brompton Hospital for Consumption. (9) An investigation on the relative importance of certain types of body-cells in defence against the tubercle-bacillus, and the effect of tuberculin and other remedial agents on their activities, by Dr. J. Hiller, pathologist to the General Hospital, Birmingham.

DR. D. EDGAR FLINN, medical inspector under the Local Government Board of Ireland, and Dr. W. J. Howarth, M.O.H., have been elected Fellows of the Royal Sanitary Institute.

A SPECIAL meeting of the Council of the Charity Organisation Society will be held on Monday, June 28, at 4.30 P.M., at Denison House, Vauxhall Bridge Road, S.W., when Miss Amy Hughes, of the Queen Victoria Jubilee Nurses' Institute, will read a paper on "Provident Nursing." Sir Alfred Lyall, G.C.I.E., K.C.B., will take the chair, and the Right Rev. the Bishop of Islington will speak.

IT is announced that Mr. John Beresford Leathes, M.B., B.Ch.Oxon, F.R.C.S.Eng., Lecturer on Physiology at St. Thomas's Hospital Medical School, has been appointed Professor of Chemical Pathology in the University of Toronto. Mr. Leathes, in addition to his appointment at St. Thomas's Medical School, has also been in charge of the Laboratory for Pathological Chemistry at the Lister Institute.

### THE CONGRESS ON ALCOHOLISM.

The forthcoming Twelfth International Congress on Alcoholism, to be held this year in London from July 18 to 24, has already been announced in our columns. We have now received a draft programme of the proceedings, and other particulars. The Congress, of which H.R.H. the Duke of Connaught is honorary president, and Lord Weardale acting-president, proposes to consider the causation, prevention and cure of alcoholism, from their individual and national aspects, by the light of research and experience in the problem of inebriety. On Sunday, July 18, the Official Sermon will be preached by the Bishop of Croydon at the afternoon service in St. Paul's Cathedral. The headquarters of the Congress will be the Imperial Institute, South Kensington, while the Sittings will be held close by, in the Kensington Town Hall, at the Imperial Institute, and at the Victoria and Albert Museum. On the Monday morning the Inaugural Address will be given by the acting-president, and in the afternoon the Exhibition will be opened by the Dean of Hereford, Chairman of the Conveners, while the evening will be devoted to the Official Reception. During the week a Reception of medical members by the British Medical Temperance Association will be held at the London Temperance Hospital. The scientific side of the Congress will occupy Tuesday, Wednesday, and Friday of the week. Papers will be read by Prof. Taav. Laitinen, M.D., on "The Influence of Alcohol on Immunity," and "A Further Contribution to the Study of the Influence of Alcohol on the Degeneration of Human Offspring"; Dr. A. Holitscher, Karlsbad, on "Alcohol in Lobar Pneumonia and Enteric Fever" and "The Consumption of Alcohol in Hospitals and Asylums"; Dr. Legrain, Paris, on "Alcohol and Insanity," and Dr. Stein, Budapest, on "Alcohol and the Nervous System." Amongst the papers to be read by British scientists will be "Alcohol and Temperance," by Professor Sims Woodhead, M.D., Cambridge; "The Action of Alcohol on Muscular and Mental Fatigue," by Dr. W. H. R. Rivers, F.R.S., Cambridge; "The Resistive Power of the Brain Against Alcohol," by Professor Clouston, M.D., Edinburgh; and "The Effects of Alcohol on the Nervous System as Exhibited in Hospital and Asylum Practice," by Dr. F. W. Mott, F.R.S., London. The treatment, prophylactic as well as remedial, of the diseases of alcoholism will be discussed on the last two days. The papers will include "Treatment of the Criminal Inebriate," by Professor Aschaffenburg of Cologne, and "Legislation for the Inebriate," by Dr. R. W. Branthwaite, H.M. Inspector under the Inebriate Acts.

